# Nutrients, surfactants, and aeration in constructed wetlands affect bacterial persistence and metabolic activity during the remediation of crude oil-contaminated water

**DOI:** 10.1186/s40643-024-00757-5

**Published:** 2024-04-20

**Authors:** Amer Jamal Hashmat, Muhammad Afzal, Samina Iqbal, Imran Amin, Carlos Alberto Arias, Hans Brix, Imran Zafar, Sania Riaz, Rizwan ur Rehman, Ahmad Mohammad Salamatullah, Gezahign Fentahun Wondmie, Mohammed Bourhia

**Affiliations:** 1 National Institute for Biotechnology and Genetic Engineering College, Pakistan Institute of Engineering and Applied Sciences (NIBGE-C, PIEAS), Faisalabad, Punjab 38000 Pakistan; 2 Centre for Water Technology (WATEC), Aarhus University, Aarhus C, 8000 Denmark; 3 Department of Bioinformatics and Computational Biology, Virtual University Pakistan, Lahore, Pakistan; 4 Faculty of Health and Life Sciences, Department of Bioinformatics and Biosciences, Capital Territory, Capital University of Science and Technology, Zone V, Kahuta Road, Islamabad, Pakistan; 5 Department of Food Science & Nutrition, College of Food and Agricultural Sciences, King Saud University, 11, P.O. Box 2460, Riyadh, 11451 Saudi Arabia; 6 Department of Biology, Bahir Dar University, P.O. Box 79, Bahir Dar, Ethiopia; 7 Laboratory of Biotechnology and Natural Resources Valorization, Faculty of Sciences, Ibn Zohr University, Agadir, 80060 Morocco; 8 Department of Biology, Aarhus University, Aarhus, Denmark

**Keywords:** Crude oil, Constructed wetlands, Bacterial persistence, Metabolic activity, Gene abundance and expression

## Abstract

The use of constructed wetlands (CWs) is one of the best options to treat wastewater. In CWs, microorganisms play a major role in the degradation of organic pollutants but the concentration of nutrients, surfactant, and aeration (NSA) in oil-contaminated water is one of the factors that affect the persistence and metabolic functioning of hydrocarbon-degrading microorganisms. In the present investigation, the influence of the addition of NSA on the persistence of the augmented bacteria, copy of (alkane hydroxylase gene) *alkB* gene, and its expression level in the water, soil, and plants of CWs were evaluated. The CWs mesocosms were developed by the vegetation of *Typha latifolia* and *Cyperus laevigatus* and inoculated with the bacterial consortium (*Pseudomonas putida* TYRI39, *Acinetobacter junii* TYRH47, *Acinetobacter* sp. CYRH17, *Pseudomonas* sp. CYSI27, and *Pseudomonas* sp. TYRH42). The mesocosms were provided with nutrients (20 mg l^− 1^ N, 2.6 mg l^− 1^ P, and 16.4 mg l^− 1^ K) in liquid form, surfactant Tween-20 (0.2%, v/v) in liquid form, and aeration (≥ 7.0 ± 1 mg l^− 1^ DO) using aeration pump. The addition of NSA in CWs enhanced the persistence and metabolic functioning of the inoculated bacteria in the water, rhizospheric soil, and plants. The maximum hydrocarbon removal (97%) was observed in the water treated by CWs having *C. laevigatus*, bacteria, and NSA, and it is correlated with the copy numbers of *alkB* and its expression level. The application of NSA in CWs not only improved bacterial persistence and catabolic gene expression but also increased plant development and hydrocarbon removal.

**Supplementary Information:**

The online version contains supplementary material available at 10.1186/s40643-024-00757-5.

## Introduction

The discharge of hydrocarbon-enriched wastewater into the environment is one of the main environmental issues with the increasing extraction, refining, and transportation of petroleum oil and its products in the world (Vymazal et al. 2021). The hydrocarbon-enriched wastewater is posing terrible effects such as genetic mutations, immunotoxicity, teratogenicity, carcinogenesis, and high bioaccumulation, impacting the health of living organisms and negatively impacting ecosystems and microbial communities (Salari et al. 2022). The discharge of hydrocarbon-enriched wastewater is an environmental challenge and causes organic pollution, and soil and aquifer contamination ultimately affecting the agricultural land and groundwater quality (Afzal et al. 2019; Wu et al. 2021). Organic chemicals including hydrocarbons are used by microorganisms during their metabolism or co-metabolism, and broken into nontoxic products like H_2_O, CH_4,_ and CO_2_ (Gu 2021).

Among the existing wastewater remediation approaches, the application of CWs has been proven more sustainable and cost-effective approach for the treatment of hydrocarbon-enriched wastewater than traditional wastewater remediation strategies (Kataki et al. 2021; Moreira and Dias 2020). Recently, the inoculation of bacteria, having hydrocarbon-utilizing and plant growth-enhancing activities in CWs has been proposed to enhance their wastewater treatment efficacy (Chen et al. 2022). The bacteria contribute to the mineralization of hydrocarbons (Hashmat et al. 2019; Yousaf et al. 2010), reduce toxicity, and increase plant development (Rehman et al. 2019). These bacteria colonize the soil and plant and mineralize organic substances including hydrocarbons (Yousaf et al. 2010). The performance of plant-bacteria synergism to mineralize hydrocarbons depends on the presence and activity of the bacteria having specific genes for the utilization of hydrocarbons (McGenity et al. 2012).

The hydrocarbon-polluted wastewater and soil are usually deficient in nitrogen and phosphorus and this deficiency inhibits plants and their associated microbial community growth and their hydrocarbon-utilizing capability (Wang et al. 2022). The addition of nutrients in oil-polluted soil and water improves microbial growth and activity and ultimately enhances the degradation of hydrocarbons (Arslan et al. 2017). Hydrocarbons are hydrophobic and their less bioavailability also reduces the chances of their microbial degradation via controlling gene side (Fenibo et al. 2023; Hashmat et al. 2022). The addition of surfactants in soil and water enhances the bioavailability of hydrocarbons and their uptake and degradation by the plants and their associated microbes (Roodbari et al. 2016). Aeration also enhances microbial growth and activity in the water (Feng et al. 2021; Mohammed Abdulredha 2018). Moreover, the effect of the addition of nutrients, surfactants, and aeration (NSA) on the proliferation and catabolic activity of the augmented bacteria in the soil and plants of CWs has not been studied earlier (Bano et al. 2023; Haider et al. 2023).

The objective of the present investigation was to assess the impact of NSA on the abundance and metabolic activity of the augmented hydrocarbon-degrading bacteria in different compartments (water, soil, and plant) of CWs. Moreover, hydrocarbon degradation, and plant growth parameters were determined.

## Materials and methods

### Bacterial strains

The bacteria used in this research work were previously isolated (Supplement Table *S1*) from the plants grown in CWs having crude oil (Hashmat et al. 2019). These bacterial strains were *Pseudomonas putida* TYRI39 (KF478204), *Acinetobacter junii* TYRH47 (KJ620859), *Acinetobacter* sp. CYRH17 (KJ620864), *Pseudomonas* sp. CYSI27 (KF478200), and *Pseudomonas* sp. TYRH42 (KF478206). These were selected because they were competent to utilize different types of hydrocarbons present in crude oil and possessed the alkane hydroxylase (*alkB*) gene. Moreover, these strains possessed plant growth-promoting traits, e.g., indole acetic acid (IAA) and siderophore production, and Zn- and P-solubilization. In brief, these bacteria were cultured individually at 37 °C in Luria Bertani broth having 2% (v/v) diesel. The cells were collected by centrifugation at 10,000 (x g) and finally, the bacterial consortium was prepared by mixing bacterial suspension in equal proportion (1:1:1:1:1) having about 10^9^ cells ml^− 1^ of each strain (Sutton 2011).

### Experimental setup

The CW mesocosms were developed as described earlier (Hashmat et al. 2019). Briefly, each CW mesocosm was made from a plastic tank having a tray filled with coconut shavings, coarse gravel, fine sand, and loamy soil. The seedlings of different wetland plants *Typha latifolia* (T1-T3) and *Cyprus laevigatus* (T4-T6) were vegetated to develop CWs mesocosms. The plants were allowed to grow for two months. The whole mesocosm system was sterilized with bleach (5%, v/v), and washed with distilled water for three times. The crude oil was spiked (2%, v/v) in the CWs. Each specific CW mesocosm was augmented with 100 ml (10^9^ cfu ml^− 1^) of the prepared bacterial consortium. Different treatments are described in Table 1. The mesocosm CWs were externally provided with optimum concentration of nutrients, i.e., NPK (20 mg l^− 1^ N, 2.6 mg l^− 1^ P, and 16.4 mg l^− 1^ K) in liquid form, surfactant (Tween- 20 with a concentration of 0.2%, v/v) in liquid form, and aeration (≥ 7.0 ± 1 mg l^− 1^ DO) using aeration pump (Hashmat et al. 2022). The CW mesocosms were operated in a batch mode with 60 60-day retention time.

**Table 1:** Design of the experiment

Treatment	Experimental design
Control (C1)	Mesocosm with vegetation, without crude oil and bacterial augmentation
Control (C2)	Mesocosm having crude oil without vegetation and bacterial augmentation
T1	Crude oil spiked CWs vegetated with *T. latifolia* (without bacterial augmentation)
T2	Crude oil spiked CWs vegetated with *T. latifolia*, and augmented with selected bacteria
T3	Crude oil spiked CWs vegetated with *T. latifolia*, augmented with selected bacteria and externally supplied with nutrients, surfactant and aeration
T4	Crude oil spiked CWs vegetated with *C. laevigatus* (without bacterial augmentation)
T5	Crude oil spiked CWs vegetated with *C. laevigatus*, and augmented with selected bacteria
T6	Crude oil spiked CWs vegetated with *C. laevigatus*, augmented with selected bacteria and externally supplied with nutrients, surfactant and aeration

### Plant growth determination

Seedlings of native wetland plants (*C. laevigatus and T. latifolia*) were collected from local drains, ponds, and the vicinity of oil drilling pits. Initially, the seedlings and/or cuttings were grown in pots for acclimatization till the achievement of suitable heights. The seedlings having almost similar weight, size, and height were used in the experiments. The plants were then used in different treatments and were harvested at the end of the experiment (day 60), and roots shoot lengths, and biomass were measured in all the treatments using a ruler and weighing balance respectively as explained earlier (Hashmat et al. 2019).

### Determination of residual amount of hydrocarbons

The water samples were collected from all the mesocosms and analyzed for the residual amount of hydrocarbons by the gravimetric method using n-hexane as solvent as explained earlier (Rehman et al. 2019).

### Isolation and quantification of the augmented bacteria

The quantification of the applied bacteria in the root, shoot, and rhizospheric soil of *T. latifolia* and *C. laevigatus* was carried out at three different stages of the experiment (day 1, day 30, and day 60). The quantification of the bacteria was also conducted in the water samples collected from the mesocosms. Briefly, the water and suspension of the rhizospheric soil, root, and shoot were plated on M9 medium (Life Technologies Catalog No. A1374401, supplemented with various amino acids and carbon sources in ready-to-use liquid form) amended with 1% diesel, incubated overnight, and then the individual colonies were counted by digital colony counter (Quebac, Hach).

### Quantification of gene (*alkB*) abundance and expression

The load of the *alkB* and its expression level were quantified in the water, soil, and plants at three different stages of the experiment (day 1, day 30, and day 60) as explained earlier (Afzal et al. 2012). The total DNA and RNA were extracted from the water, rhizospheric soil, and the roots and shoots. Real-time PCR was used to determine the copy numbers of the *alkB* gene using the template of DNA and cDNA.

### Statistical analysis

The plant growth parameters, CFU, level of gene abundance and expression, and the residual number of hydrocarbons were analyzed using Minitab statistical software (www.minitab.com) as per the method of (Ain et al. 2024). One-way analysis of variance (ANOVA) was used for the comparisons between treatments (Ahmad et al. 2023).

## Results

### Plant growth

**Fig. 1:**
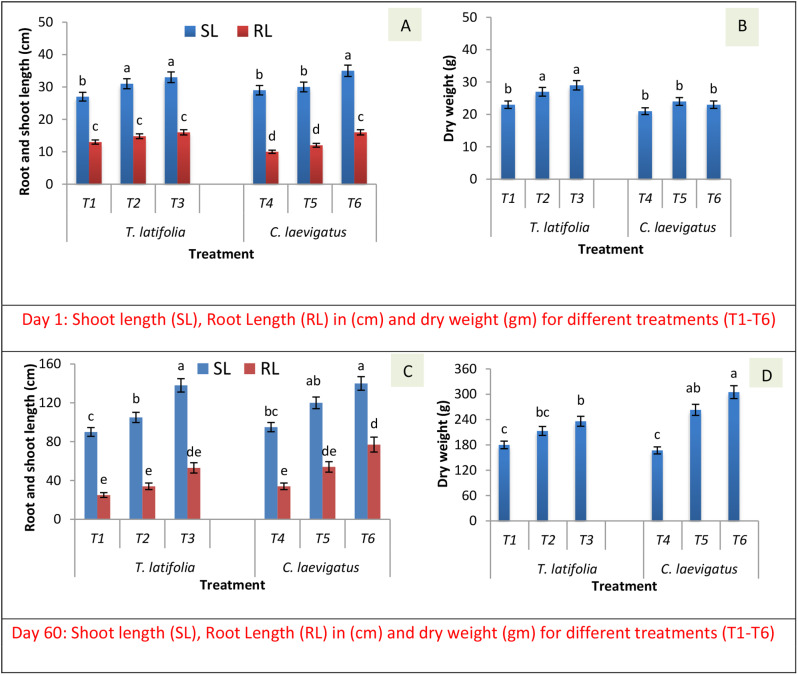
The effect of intensification (augmentation of bacteria, nutrients, surfactant and aeration) on the growth of wetland plants (*T. latifolia* and *C. laevigatus)*. The root length (RL), shoot length (SL) and dry weight (DW) at day 1 (Figs. **A** and **B**), and at day 60 (Figs. **C** and **D**). (T1 and T4): The constructed wetlands (CWs) planted with *T. latifolia* and *C. laevigatus* respectively without bacterial augmentation. (T2 and T5): The CWs planted with *T. latifolia* and *C. laevigatus* respectively augmented with selected bacteria possessing *alkB* genes. (T3 and T6): The CWs planted with *T. latifolia* and *C. laevigatus* respectively augmented with selected bacteria possessing *alkB* genes and provided with nutrients, surfactant and aeration. The error bar indicates standard error among three replicates. The values with same letter are not different at 5% significance level. The analysis of variance (ANOVA) was used for evaluations amongst different treatments

The wetland plant (*T. latifolia* and *C. laevigatus*) growth data in terms of root length, shoot length, and dry weight were recorded on day 1 and day 60. The effect of intensification in wetland on the plant growth parameters was assessed by employing the influence of bacterial augmentation alone as well as the combined effect of bacterial augmentation and NSA application on the growth of the plants vegetated in the CWs (Fig. 1). There was a sequential growth of *T. latifolia* and *C. laevigatus* from day 1 to day 60. At day 1, there was a non-significant (*p* < 0.05) difference in the root length (RL), shoot length (SL), and dry weight (DW) of the plants vegetated in CWs (T1-T6) (Fig. 1A and B), showing that the plants chosen at the beginning of the experiment were of almost similar age, size and weight. There was an increase in the development of the plants during 60 days of their plantation in CWs having crude oil (2%, v/v). The bacterial augmentation in CWs (T2 and T5) significantly (*p* < 0.05) improved the SL (14–35%), RL (26–32%), and DW (18–24%) of both plants compared to that observed of the plants vegetated in the CWs without bacterial augmentation (T1 and T4) (Fig. 1C and D).

The addition of NSA in bacterial-augmented CWs (T3 and T6) further enhanced the growth (12–14%, 9–13%, and 10–24% RL, SL, and DW, respectively) of the plants (Fig. 1C and D). Between the two plants, the maximum increment in the SL (49%), RL (45%), and DW (48%) were found in *C. laevigatus* augmented with the bacteria and aided with NSA (T6) than that of the plant without bacterial augmentation and NSA application. The growth of both plants was in the following order: without bacterial augmentation < bacterial augmentation < bacterial augmentation and provision of NSA.

### Persistence and metabolic activity of the bacteria

**Fig. 2:**
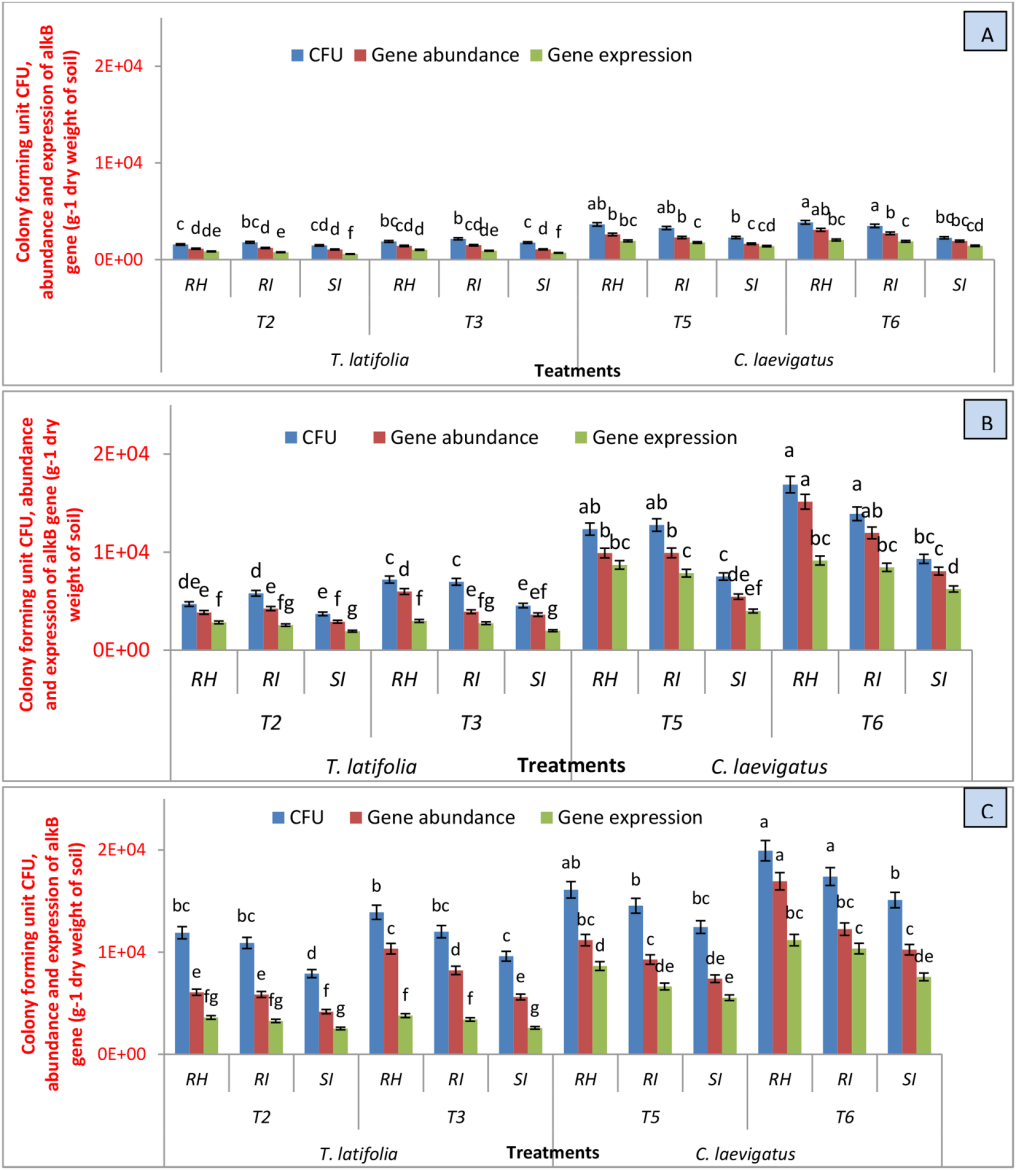
Bacterial colonization (CFU g^− 1^ dry weight of soil/plant material), gene abundance (copies of *alkB* g^− 1^ dry weight of soil/plant material) and gene expression (transcripts level of *alkB* gene g^− 1^ dry weight of soil/plant material) present in rhizosphere (RH), root interior (RI) and shoot interior (SI) of *T. latifolia* and *C. laevigatus* at day 1 (**A**), day 30 (**B**) and day 60 (**C**) in different treatments. crude oil spiked CWs vegetated with *T. latifolia* and inoculated with the bacteria (T2); crude oil spiked CWs vegetated with *T. latifolia* and inoculated with the bacteria and supplied with nutrients, surfactant and aeration (T3); crude oil spiked CWs vegetated with *C. laevigatus* and inoculated with the bacteria (T5), crude oil spiked CWs vegetated with *C. laevigatus* and inoculated with the bacteria and supplied with nutrients, surfactant and aeration (T6). The values with same letter are not different at 5% significance level. The analysis of variance (ANOVA) was used for evaluations amongst different treatments. (Y axis shows value in e^x^)

The bacterial persistence, copy numbers of the *alkB* gene, and its expression level were monitored in the water, soil, and plants (Fig. 2). In the water, rhizospheric soil, root, and shoot, the increase in CFU and copy numbers of *alkB* and its expression level from day 1 to day 60 revealed that the inoculated bacteria tended to proliferate and were also metabolically active. The inoculated hydrocarbon-degrading bacterial population and activity in the mesocosms applied with NSA (T3 and T6) were significantly (*p* < 0.05) higher compared to the mesocosms without the addition of NSA (T2 and T5). Between the two plants, more bacterial persistence and activity were observed in the rhizospheric soil and endosphere of *C. laevigatus.*

More bacterial persistence and the numbers of *alkB* gene and its expression level were observed in the rhizospheric soil than in the roots and shoots of both plants. Between the two plants, *C. laevigatus* hosted comparatively higher numbers of the inoculated bacteria, *alkB* gene, and its expression level in its rhizospheric soil and biomass which was significantly higher (*p* < 0.05) compared to that of *T. latifolia* (T3) (Fig. 2C).

### Hydrocarbon degradation

The effect of inoculation of bacteria, and the combined effect of inoculation of bacteria and NSA addition in CWs on total hydrocarbons (THs) reduction were recorded (Table 2). The residual amount of THs was measured at three different stages, day 1, 30, and 60. There was a gradual decrease in the THs contents in the water of all the treatments including control from day 1 to day 60. There was more THs removal (26.8–32.8% and 33.5–68%, at day 30 and day 60, respectively) in the water of CWs vegetated with *T. latifolia* and *C. laevigatus* (T1 and T4) than in the water of the mesocosms without vegetation and bacterial inoculation. The CWs (T2 and T5) vegetated with *T. latifolia* and *C. laevigatus* and augmented with the bacterial strains showed more THs degradation (43.8–71.9% and 57.5–80.3%, at day 30 and day 60, respectively) than in the water of the CWs mesocosms having only vegetation.

**Table 2:** Effect of bioaugmentation and addition of nutrients, surfactant, and aeration on hydrocarbon degradation in CWs vegetated with *T. latifolia* and *C. laevigatus*

Treatment	THs (g l^− 1^)	THs (g l^− 1^)	THs (g l^− 1^)
	Day 0	Day 30	Day 60
C1	ND	ND	ND
C2	20	18.3 (0.46)^e^	16.8 (0.42)^f^
T1	20	14.6 (0.76)^d^	13.3 (0.69)^e^
T2	20	11.2 (0.75)^c^	5.6 (0.38)^c^
T3	20	3.4 (0.57)^a^	1.7 (0.57)^a^
T4	20	13.4 (0.27)^d^	6.4 (0.27)^d^
T5	20	8.4 (0.47)^b^	3.9 (0.13)^b^
T6	20	1.7 (0.35)^a^	0.6 (0.11)^a^

Control (C1) mesocosm with vegetation and without crude oil and bacterial inoculation, control (C2) mesocosm without vegetation and bacterial inoculation, crude oil spiked CWs with *T. latifolia* vegetation and without bacterial augmentation (T1), crude oil spiked CWs vegetated with *T. latifolia* and inoculated with the bacteria (T2), crude oil spiked CWs vegetated with *T. latifolia* and inoculated with the bacteria and supplied with nutrients, surfactant and aeration (T3), crude oil spiked CWs vegetated with *C. laevigatus* and without bacteria(T4), crude oil spiked CWs vegetated with *C. laevigatus* and inoculated with the bacteria (T5), crude oil spiked CWs vegetated with *C. laevigatus* and inoculated with the bacteria and supplied with nutrients, surfactant and aeration (T6). The same letters are not significantly different at a 5% level of significance, *n* = 3; the standard error of three replicates is given in parenthesis. Where ND is not detected

The CWs mesocosms having bacterial augmentation and provision of NSA exhibited significantly (*p* < 0.05) more THs reduction in the water of T3, i.e., 83 and 91.4%, and T6, i.e., 91.5 and 97%, at day 30 and day 60, respectively, than in the water of CWs mesocosms having only bacterial inoculation. The CWs vegetated with *T. latifolia* (T3) and *C. laevigatus* (T6) and inoculated with the bacteria and supplied with NSA have more THs reduction, 20 and 40%, and 16 and 33%, at day 30 and day 60, respectively, than the treatments T2 and T5 (only bacterial inoculation). The maximum THs reduction (97%) was achieved in the CWs vegetated with *C. laevigatus*, inoculated with *alkB-possessing* bacteria, and externally aided with NSA (T6).

## Discussion

In this study, there was significantly less growth of the plants in CWs having crude oil compared to the plants grown in tap water (control). On the contrary, the bacterial augmentation in CWs (T2 and T5) significantly improved shoot and root lengths and dry weight of both wetland plants (*T. latifolia* and *C. laevigatus*) compared to that observed in the CWs without augmentation of selected bacterial strains possessing *alkB* genes (mentioned in Sect. 2.1) in treatments (T1 and T4). Several earlier studies reported that bacterial augmentation in soil and water may improve plant growth (Rehman et al. 2019). Bacteria degrade the organic compounds reduce their toxicity and ultimately improve plant growth (Masciandaro et al. 2013).

The external supplement of NSA in bacterial-augmented CWs (T3 and T6) enhanced the growth of the plants, compared to the treatment (T2 and T5) with just bacterial augmentation and NSA was not supplied. This may be because the nutrients are required by plants to perform their photosynthesis and other metabolic processes (Gkorezis et al. 2016). The surfactant decreases the viscosity of the crude oil and improves bioavailability by forming a complex oil-in-water system in which the oil can become easily available to roots and bacteria (Xu et al. 2018).

Aeration enhances microbial growth and hydrocarbon degradation activity (Syafruddin et al. 2010). Similar findings were also observed in our previous investigations that the addition of NSA in CWs has positive effects on plant development (Hashmat et al. 2022). Moreover, in the present study, the maximum increment in the SL, RL, and DW was found in *C. laevigatus* inoculated with the bacteria and aided with NSA (T6) compared to *T. Latifolia*. This may be because different plants exhibit different growth responses in the presence of crude oil (Fatima et al. 2016; Hashmat et al. 2019).

In the present study, the colony forming unit (CFU), the *alkB* gene abundance, and expression were determined at 3-time intervals (day 1, day 30, and day 60) in the rhizospheric soil, plant roots, and shoots grown in intensified wetlands. It was revealed that the inoculated bacteria tended to proliferate and were metabolically active (Fig. 2). Despite that, the inoculated hydrocarbon-degrading bacterial population and activity in the mesocosms aided with NSA (T3 and T6) were significantly (*p* < 0.05) higher compared to the wetland mesocosms without the addition of NSA (T2 and T5). These observations indicated that the addition of NSA in CWs positively influenced the proliferation of the selected inoculated bacteria (possessing *alkB* genes). Previous investigations (Xu et al. 2018) also described that the addition of surfactant and aeration may propagate specific bacterial strains.

The maximum bacterial persistence and copy numbers of the *alkB* gene and its expression level were observed in the rhizospheric soil, followed by roots and shoots of *C. laevigatus* (T6) which were significantly higher (*p* < 0.05) compared to that of *T. latifolia* (T3) (Fig. 2C). The more population and metabolic activity of the bacteria in the rhizospheric soil than in the root and shoot may be because plants provide more nutrients through their root’s exudates in the rhizospheric soil for microbial proliferation than in the roots and shoots. Similarly, specific bacteria can successfully penetrate the host plant and thus their population may increase in the plant tissues (Afzal et al. 2019; Zhang et al. 2014).

In this study, it was found that there is a higher number of bacteria (possessing *alkB* gene) with high CFU value and high level of expression (Gene copy number) in the treatments (T3 and T6) where intensification in wetlands was carried out by NSA compared to (T2 and T5). This might be because we have externally supplied nutrients, surfactant, and aeration, which are otherwise limiting factors for the growth of the bacterial population. These findings are from earlier studies (Arslan et al. 2017) where nutrients were found to be the limiting factor for the bacterial population and their existence in the roots and shoots of the plants. In this study, bacterial colonization in the crude oil-contaminated water revealed that the inoculated bacteria utilized hydrocarbons as a source of energy and propagated in an oil-polluted environment.

There prevalence of *alkB-possessing* bacteria was higher in the rhizospheric soil and endosphere of the *C. laevigatus* plant compared to *T. latifolia.* This variance can be described by the difference in the composition of root’s released compounds of the two plant species (Burgmann et al. 2005). Moreover, different plant species have different numbers and colonization patterns of symbiotic bacteria in their rhizospheric soil and vegetative parts (Tara et al. 2014). Our data showed that *C. laevigatus* is a comparatively more suitable host plant than *T. latifolia* for attracting hydrocarbon-degrading bacterial communities.

The total hydrocarbon (THs) removal was significantly (*p* < 0.05) low in the control experiments (wetlands without vegetation and bacterial inoculation) compared to the wetlands vegetated with *T. latifolia* and *C. laevigatus* and augmented with *alkB* gene possessing specific bacteria. A slight reduction in THs in the control experiment (after 60) days may be due to the degradation of hydrocarbons by indigenous bacteria and sunlight, and the evaporation of volatile compounds as established in a previous study (Afzal et al. 2012). Bacterial inoculations (Table 2) in the CWs (T2 and T5) vegetated with *T. latifolia* and *C. laevigatus* caused more THs degradation than in the CWs without bacteria inoculation (T1 and T4). These results indicate that the plant-bacteria partnership resulted in more hydrocarbon degradation, this is in alignment with previous findings (Afzal et al. 2019; Fatima et al. 2016).

Moreover, in the present study, significantly (*p* < 0.05) more THs reduction was achieved in T3 and T6 than in control and other treatments. These results exhibited that the CWs vegetated with *T. latifolia* (T3) and *C. laevigatus* (T6), inoculated with the bacteria and supplied with NSA have more THs reduction than in the treatments T2 and T5 (vegetated and bacterial inoculated, but without NSA). The maximum THs reduction (97%) was achieved in the CWs vegetated with *C. laevigatus*, inoculated with bacteria, and externally aided with NSA (T6). Several previous investigations also stated that the addition of NSA enhanced the degradation of hydrocarbons (Zada et al. 2021). It might be because the addition of nutrients and aeration enhances microbial growth and hydrocarbon degradation, whereas surfactant enhances the bioavailability of the hydrocarbon in the soil and water (Fatima et al. 2016).

## Conclusions

The addition of NSA in CWs enhanced bacterial persistence and metabolic activity in the water, rhizospheric soil, and vegetative parts of the plants, and ultimately plant development, and hydrocarbon removal. Between the two plants, more bacterial growth and activity were seen in the rhizospheric soil and vegetative parts of *C. laevigatus*, this plant also exhibited more hydrocarbon degradation.

### Electronic supplementary material

Below is the link to the electronic supplementary material.

Supplementary Material 1

## Data Availability

Data will be available upon request from the corresponding author.
