# Supplementary figures and images for: Nutrients, surfactants, and aeration in constructed wetlands affect bacterial persistence and metabolic activity during the remediation of crude oil-contaminated water
Source: Bioresour Bioprocess. 2024 Apr 20;11(1):40. doi: 10.1186/s40643-024-00757-5 (PMC11031516; doi:10.1186/s40643-024-00757-5)

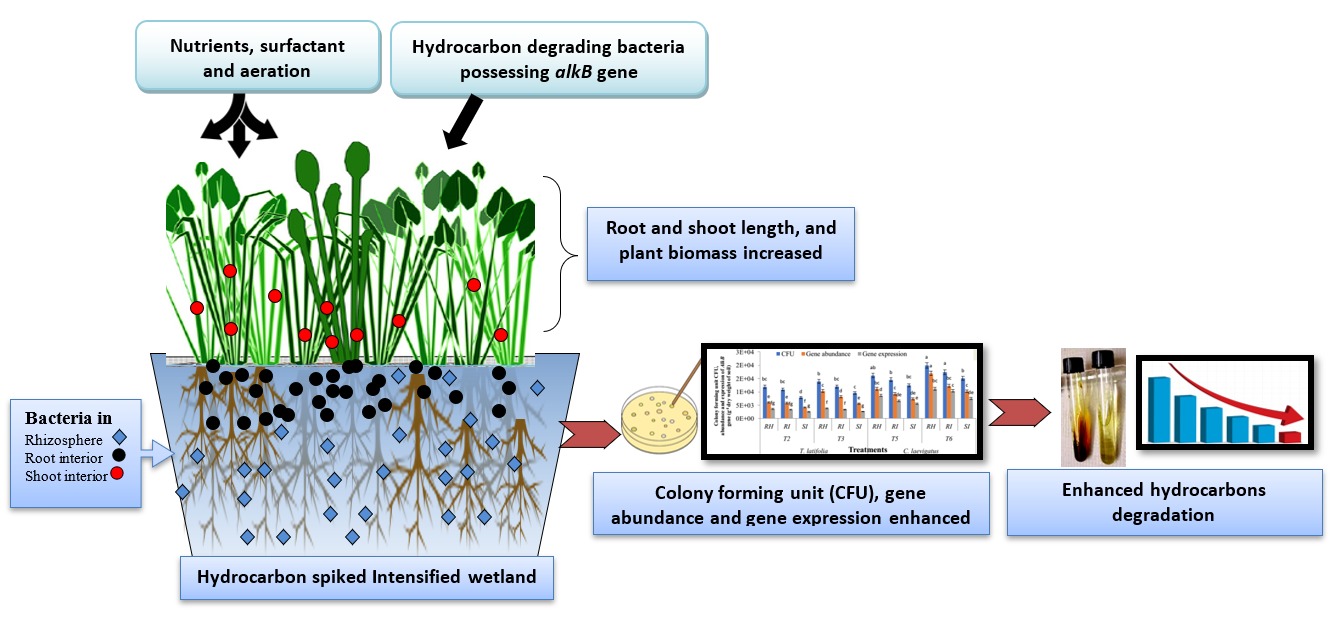

Supplement: Supplementary file 1 — Supplementary Material 1 [file 40643_2024_757_MOESM1_ESM.jpeg]
